# Development of a prognostic prediction model and visualization system for autologous costal cartilage rhinoplasty: an automated machine learning approach

**DOI:** 10.3389/fsurg.2025.1594514

**Published:** 2025-10-02

**Authors:** Aihemaitijiang Niyazi, Tilimanjiang Tuohuti, Xu Nannan, Dawuli Shalimujiang, Yang Zhao

**Affiliations:** 1 Department of Burns, The First Affiliated Hospital of Xinjiang Medical University, Urumqi, Xinjiang, China; 2 Department of Plastic Surgery, Xi Jing Hospital, Air Force Medical University, Xi’an, Shaanxi, China; 3 YanTa Aesthetic Clinic, MingNanDuoMei Medical Cosmetic Co., Ltd., Xi’an, Shaanxi, China

**Keywords:** autologous costal cartilage rhinoplasty, automated machine learning, prognostic modeling, explainable AI, clinical decision support system

## Abstract

**Objective:**

To develop an automated machine learning (AutoML)-based prognostic prediction model and visualization system for autologous costal cartilage rhinoplasty (ACCR), addressing the clinical challenges of postoperative complications and satisfaction disparity.

**Methods:**

A retrospective cohort of 447 ACCR patients (2019–2024) was analyzed, integrating 20+ parameters spanning biological, surgical, and behavioral domains. We proposed an improved metaheuristic algorithm (INPDOA) for AutoML optimization, validated against 12 CEC2022 benchmark functions. Bidirectional feature engineering identified critical predictors, and SHAP values quantified variable contributions. A MATLAB-based clinical decision support system (CDSS) was developed for real-time prognosis visualization.

**Results:**

The INPDOA-enhanced AutoML model outperformed traditional algorithms, achieving a test-set AUC of 0.867 for 1-month complications and *R*^2^ = 0.862 for 1-year Rhinoplasty Outcome Evaluation (ROE) scores. Key predictors included nasal collision within 1 month, smoking, and preoperative ROE scores. Decision curve analysis demonstrated a net benefit improvement over conventional methods. The CDSS reduced prediction latency.

**Conclusion:**

This study establishes the first AutoML-driven prognostic framework for ACCR, effectively bridging the gap between surgical precision and patient-reported outcomes. Its integration of dynamic risk prediction and explainable AI offers a paradigm for aesthetic surgical decision-making.

## 1 Introduction

Rhinoplasty, as one of the most widely performed aesthetic surgeries worldwide, has evolved with dual objectives of morphological aesthetics and functional reconstruction. Autologous costal cartilage rhinoplasty (ACCR), since its introduction in the 1990s (1), has become the gold standard for correcting severe saddle nose deformities, post-traumatic nasal defects, and revision cases following failed prior surgeries. Compared to silicone or expanded polytetrafluoroethylene (ePTFE) implants, ACCR offers three core advantages: (1) non-immunogenicity due to autologous tissue utilization significantly reduces infection risk (2); (2) three-dimensional sculptability of cartilage enables complex nasal tip reconstruction (3); and (3) biomechanical compatibility with host tissues ensures long-term structural stability (4). According to the International Society of Aesthetic Plastic Surgery (ISAPS), ACCR adoption in Asia has surged by 37% over the past 5 years, establishing it as the fastest-growing subspecialty in rhinoplasty (5).

Nevertheless, ACCR remains challenged by unpredictable postoperative outcomes. Multicenter cohort studies (6–8) reveal a 12-month complication profile encompassing variable graft resorption rates (5.8%–21.4%), dorsal contour irregularity (7.3%), incision scar hypertrophy (9.5%), and hematoma (1.2%), with 11.6% of cases requiring revision surgery. Notably, significant discrepancies persist between patient-reported satisfaction and surgeon assessments (9), underscoring systemic deficiencies in existing prognostic evaluation frameworks. These limitations stem from: (1) inadequate quantification of patient-specific factor interactions, such as costal cartilage calcification (Lemon grading), nasal skin thickness/elasticity, and alar-columellar proportions; (2) intraoperative decision-making overly reliant on empirical expertise without real-time data support; and (3) misaligned risk perception in traditional physician-patient communication.

Early prognostic tools in plastic surgery—exemplified by first-generation multivariate regression models—achieved limited success. For instance, the CRS-7 complication scale for septoplasty demonstrated an AUC of 0.68 with seven clinical predictors (10), constrained by linear assumptions ill-suited for nonlinear biological systems, neglect of higher-order interactions (e.g., BMI-dependent tissue perfusion threshold effects), and inability to integrate emerging modalities like radiomics. The advent of electronic medical records (EMR) and picture archiving systems (PACS) catalyzed second-generation machine learning (ML) models, achieving breakthrough performance in facial fracture prognosis prediction (AUC = 0.81) (11) and deep learning-based pain forecasting in breast reconstruction (81.3% accuracy) (12). However, persistent limitations include: (1) manual feature engineering and hyperparameter tuning compromising reproducibility; (2) ineffective dimensionality reduction for high-parameter spaces; and (3) poor clinical translation, with most models confined to academic literature.

Automated machine learning (AutoML)—a frontier in artificial intelligence (AI)—revolutionizes medical predictive modeling through end-to-end automation (13). Key innovations include: (1) neural architecture search (NAS) outperforming manual CNN designs in classification tasks (14); (2) Bayesian optimization-driven hyperparameter tuning slashing development cycles (15); and (3) automated feature engineering modules (e.g., TPOT, Auto-Sklearn) generating high-order interaction terms. Yet, AutoML applications in aesthetic surgery remain unexplored, partly due to data heterogeneity (3D scans, tissue biomechanics, dynamic expression capture) and the imperative for interpretability to align subjective aesthetic expectations with clinical decisions.

Our study pioneers the integration of AutoML into ACCR prognosis, aiming to usher rhinoplasty into the era of predictive medicine. By establishing a risk-stratified AI framework, we seek to preempt complications, harmonize patient-surgeon satisfaction metrics, and provide actionable insights for intelligent aesthetic surgery.

## 2 Methods

### 2.1 Study population

This retrospective study received ethical approval from the Institutional Review Board of Xi Jing Hospital (Approval No. K202504-12), with informed consent waived due to the anonymized nature of the data. We analyzed 447 patients who underwent ACCR from March 2019 to January 2024 across two centers: Xi Jing Hospital (*n* = 330) and MingNanDuoMei Aesthetic Hospital (*n* = 117). The Xijing Hospital cohort (*n* = 330) demonstrated a mean age of 25.15 ± 5.32 years (range: 18–35), comprising 27 male and 303 female participants. The Mingnan Duomei Aesthetic Medical Center cohort (*n* = 112) presented comparable demographics with a mean age of 24.89 ± 6.34 years (range: 18–36), including 11 male and 101 female subjects—reflecting the predominantly female distribution characteristic of elective cosmetic procedure populations at this tertiary institution.

Inclusion criteria: (1) Primary or revision ACCR; (2) Complete 1-year follow-up data.

Exclusion criteria: (1) Age <18 years; (2) Implant removal due to dissatisfaction; (3) Pregnancy or lactation; (4) Severe cardiac/hepatic dysfunction; (5) History of cleft lip-nose repair.

### 2.2 Data collection

Data were extracted from institutional electronic medical records (EMRs) and manually cross-validated to ensure consistency. Following categorization by variable type: (1) Demographic variables: Age, sex, body mass index (BMI), and education level; (2) Preoperative clinical factors: Nasal pore size, prior nasal surgery history, and preoperative Rhinoplasty Outcome Evaluation (ROE) score (16); (3) Intraoperative/surgical variables: Surgical duration (hours) and length of hospital stay (days); (4) Postoperative behavioral/event factors: Documented occurrences within the first postoperative month, including nasal trauma (binary yes/no), antibiotic duration (categorized as <3 days/3–5 days), folliculitis, animal contact, spicy food intake, smoking, and alcohol use; (5) Outcome measures: Short-term (1 month): Composite endpoint of infection, hematoma, or graft displacement; Long-term (1 year): ROE score (range: 0–100) for cosmetic and functional assessment. Clinical correlations, data collection methodology, and implementation details are comprehensively documented in Supplementary Table S1.

### 2.3 Model development

The Xi Jing cohort was divided into training (*n* = 264) and internal test sets (*n* = 66) using an 8:2 split, while the MingNanDuoMei cohort served as an external validation set (*n* = 117). To minimize selection bias while preserving outcome distribution consistency, the Xijing Hospital cohort was partitioned into training and testing sets through stratified random sampling. Stratification criteria comprised preoperative ROE score tertiles (Low: 0–25 points; Medium: 26–35 points; High: >35 points) and 1-month complication status (yes/no). For classification modeling predicting 1-month complications, the Synthetic Minority Oversampling Technique (SMOTE) was applied exclusively to the training set to address class imbalance. Validation sets maintained original data distributions to accurately reflect real-world clinical scenarios. The proportion of missing values within the dataset was minimal (1.3%). For continuous variables (e.g., ROE score), missing values were replaced with the median of the corresponding variables; for categorical variables (e.g., comorbidity types), missing values were imputed using the mode. A 10-fold cross-validation strategy mitigated overfitting.

Automated machine learning (AutoML) framework: This study employs an AutoML framework based on optimization algorithms, integrating in-depth three synergistic mechanisms: base-learner selection, feature screening, and hyperparameter optimization. To ensure methodological rigor, the original dataset underwent stratified random assignment into training and held-out independent test sets at the experimental outset. All subsequent procedures—including feature selection, model configuration refinement, and cross-validation assessment—were strictly confined within the training subset. The framework uniformly encodes three decision spaces into a hybrid solution vector:

$$x=(\underset{\mathrm{model}\;\mathrm{type}}{\underbrace{k}}|\underset{\mathrm{feature}\;\mathrm{selection}}{\underbrace{\delta_{1},\delta_{2},\ldots ,\delta_{m}}}|\underset{\mathrm{hyper}\text{-}\mathrm{parameters}}{\underbrace{\lambda_{1},\lambda_{2},\ldots ,\lambda_{n}}})$$

Where the base-learner type is discretely defined (*k*: 1 = Logistic Regression [LR], 2 = Support Vector Machine [SVM], 3 = XGBoost, 4 = LightGBM); feature selection follows binary 0/1 encoding; and hyperparameter space adapts dynamically to the selected base model. Driven by swarm intelligence algorithms, each iteration comprises: (a) identifying the candidate base-learner per *k*-value in the solution vector; (b) extracting a feature subset via the solution vector; and (c) injecting adaptive parameters to instantiate the model. Configured model instances then undergo rigorous ten-fold cross-validation within the training set, forming a synergistic feedback loop for “architecture–feature representation–parameterization”. Synergistic optimization is governed by a dynamically weighted fitness function:

$$f(x)=w_{1}(t)\cdot \mathrm{AC}C_{\mathrm{CV}}+w_{2}\cdot \left(1-\frac{{\left\|\delta \right\|}_{0}}{m}\right)+w_{3}\cdot \exp\;(-T/T_{\max})$$

This function holistically balances three critical dimensions: predictive accuracy (ACC term), feature sparsity (ℓ₀norm), and computational efficiency (exponential decay term). Weight coefficients *α*(*t*), *β*(*t*), *γ*(*t*) adapt across iterations—prioritizing accuracy initially, balancing accuracy and sparsity mid-phase, and emphasizing model parsimony terminally [where *α*(*t*) ≈ *β*(*t*)]. Performance benchmarking includes traditional models (LR, SVM) and ensemble learners (XGBoost, LightGBM). For individual sample prediction, the AutoML model yields class probability confidence: For a new sample with feature vector *x*, the classification probability output through forward propagation is denoted as:

$$p=\sigma (w^{T}\cdot \phi (x)+b)$$

Where σ denotes the sigmoid activation $\sigma (z)=\frac{1}{1+e^{-z}}$, ϕ(x) the engineered feature transformation, *w* the output layer weight vector, and *b* the bias term.

**Figure 1:**
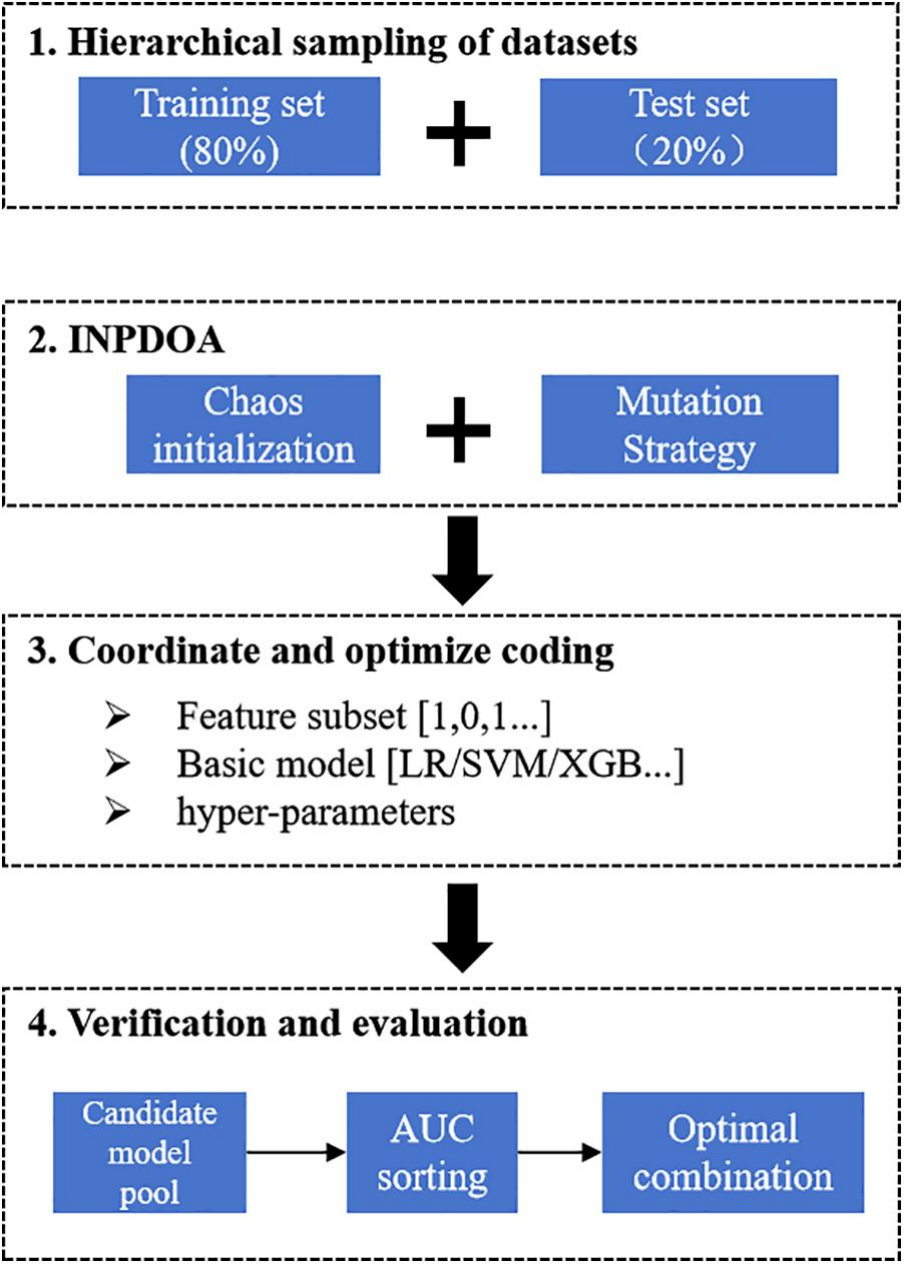
Flowchart of the AutoML.

An adaptive ensemble AutoML framework driven by the Improved Neural Population Dynamics Optimization Algorithm (INPDOA) was constructed to develop the predictive model. At the algorithmic level, we proposed an INPDOA enhanced through dual-strategy modifications to address traditional metaheuristics' susceptibility to convergence on local extrema during high-dimensional optimization. Building upon the classical Neural Population Dynamics Optimization Algorithm (NPDOA) (17), our approach: (i) reconstructs the initial population using Bernoulli mapping, and (ii) incorporates a Lévy flight random walk strategy to regulate the global-local convergence balance across individuals, thereby enhancing adaptability to complex parameter spaces (18). During model construction, the iterative INPDOA process concurrently: (i) generated binary-encoded feature subsets (where 1 = selected feature, 0 = excluded feature), and (ii) dynamically selected base learners with their hyperparameters (regularization coefficients, tree depth, and learning rate), yielding a diverse candidate model pool. Model performance was quantified via cross-validated AUC on the training set, ultimately determining the optimal combination of feature subsets, base learners, and hyperparameters. The flowchart is illustrated in Figure 1.

Benchmarking: INPDOA was validated through 30 independent runs on 12 CEC2022 benchmark functions (dimensions = 10, population = 30, iterations = 500) (19), outperforming NPDOA, genetic algorithm (GA), and whale optimization algorithm (WOA).

Model comparison: Logistic regression (LR), support vector machine (SVM), Adaboost, XGBoost, and LightGBM were evaluated. Two models were trained: Classification model: 1-month complication risk (binary outcome). Regression model: 1-year ROE score prediction.

### 2.4 Model evaluation

#### 2.4.1 Validation strategy

A progressive three-stage validation framework was implemented: initial evaluation employed tenfold stratified cross-validation within the training cohort; subsequent assessment leveraged an internally reserved test set (*n* = 66); culminating in external validation using a multicenter cohort (*n* = 117) to verify generalizability across heterogeneous clinical environments, thereby ensuring the model's robustness mirrors real-world variability.

#### 2.4.2 Performance metric system

Classification metrics: Accuracy (ACC), sensitivity (SEN), specificity (SPE), *F*1-score, AUROC, and precision-recall AUC (PR-AUC).

Regression metrics: Coefficient of determination (*R*^2^), mean squared error (MSE), root MSE (RMSE), mean absolute error (MAE), and mean absolute percentage error (MAPE).

#### 2.4.3 Quantification of clinical utility

Decision curve analysis (DCA) was applied to quantify clinical utility by computing net benefit (NB) across varied threshold probabilities. The operational formula is defined as:

$$\mathrm{NB}=\frac{\mathrm{TP}}{N}-\frac{\mathrm{FP}}{N}\times \frac{\;p_{t}}{1-p_{t}}$$

where TP denotes true positives, FP signifies false positives, *N* represents the total cohort size, and *P_t_* indicates the predefined risk threshold. Comparative assessment of model-derived NB against traditional intervention reference lines established the validated effective range for clinical decision support.

### 2.5 Explainability analysis

Our study employed SHAP (SHapley Additive exPlanations) analysis to investigate the interpretability of the predictive model. Rooted in cooperative game theory's Shapley values, the SHAP method assigns each feature an importance value, enabling precise quantification of its contribution towards model predictions. This approach explains model behavior both globally (overall patterns) and locally (prediction logic for individual instances). To comprehensively demonstrate interpretability, we generated three classes of SHAP visualizations: (1) SHAP Summary Plot: This integrates feature importance across all samples and indicates effect directionality. Each point represents a feature's SHAP value for one sample, color-coded by feature value (yellow = high, blue = low), visually depicting positive or negative associations between features and predicted outcomes. (2) SHAP Feature Importance Plot: This ranks features by their global impact on predictions based on the mean absolute SHAP value, facilitating identification of the most influential predictors. (3) SHAP Dependence Plots: Quantify the marginal effect of individual variables and reveal potential clinical decision thresholds.

### 2.6 Clinical decision support system

A MATLAB App Designer-based system was developed, integrating trained models into a user-friendly dashboard. Clinicians input patient variables to obtain real-time predictions and personalized risk mitigation strategies.

### 2.7 Statistical analysis

Continuous variables are presented as mean ± SD (one-way ANOVA for comparisons); categorical variables as frequencies (chi-square test). *P* < 0.05 defined statistical significance. Analyses were performed in MATLAB 2024b.

## 3 Results

### 3.1 Clinical characteristics and outcomes

The training set (*n* = 264) showed 26.52% (70/264) short-term adverse events (infection/hematoma/graft displacement), with preoperative and 1-year postoperative ROE scores of 31.28 ± 8.15 and 87.76 ± 18.23, respectively. Comparable trends were observed in the test set (*n* = 66: 27.27% adverse events; preoperative ROE = 30.93 ± 7.83; postoperative ROE = 87.14 ± 13.08) and external validation set (*n* = 117: 23.93% adverse events; preoperative ROE = 32.27 ± 9.05; postoperative ROE = 86.33 ± 11.63). No significant differences were observed across all variables among groups (all *P* > 0.05), confirming cohort homogeneity (Table 1).

**Table 1:** Comparison of clinical characteristics and outcome indicators of each data set.

Feature	Training set (*n* = 264)	Internal test set (*n* = 66)	Validation set (*n* = 117)	*P*-value
Clinical characteristics				
Age (years)	25.27 ± 5.13	24.66 ± 6.06	24.89 ± 6.34	0.668
Gender (male/female)	20/244	7/59	11/101	0.637
BMI (kg/m^2^)	20.27 ± 8.43	21.58 ± 7.96	20.13 ± 8.09	0.467
Education (≤high school/>high school)	236/28	57/9	106/11	0.670
Enlarged nasal pores (yes/no)	20/244	6/60	8/109	0.858
Nasal surgery history (Yes/No)	25/239	6/60	9/108	0.854
Hospital stay (≥5 days/<5 days)	111/153	32/34	40/77	0.143
Surgery duration (≥8 h/<8 h)	26/238	6/50	10/107	0.852
Preoperative ROE score	31.28 ± 8.15	30.93 ± 7.83	32.27 ± 9.05	0.477
Nasal collision within 1 month (yes/no)	16/248	6/60	8/109	0.678
Antibiotic use duration (<3 days/3–5 days)	53/211	13/53	20/97	0.789
Postoperative folliculitis (yes/no)	30/234	8/58	10/107	0.662
Animal contact within 1 month (yes/no)	40/224	11/55	16/101	0.857
Spicy food intake within 1 month (yes/no)	34/230	9/57	15/102	0.985
Smoking within 1 month (yes/no)	21/243	10/50	14/103	0.101
Alcohol consumption within 1 month (yes/no)	26/238	13/53	16/101	0.081
Outcome measures				
Poor prognosis at 1 month (yes/no)	70/194	18/48	28/89	0.839
ROE score at 1 year postoperative	87.96 ± 14.28	87.14 ± 13.08	86.33 ± 11.63	0.619

### 3.2 Algorithm optimization performance

**Figure 2:**
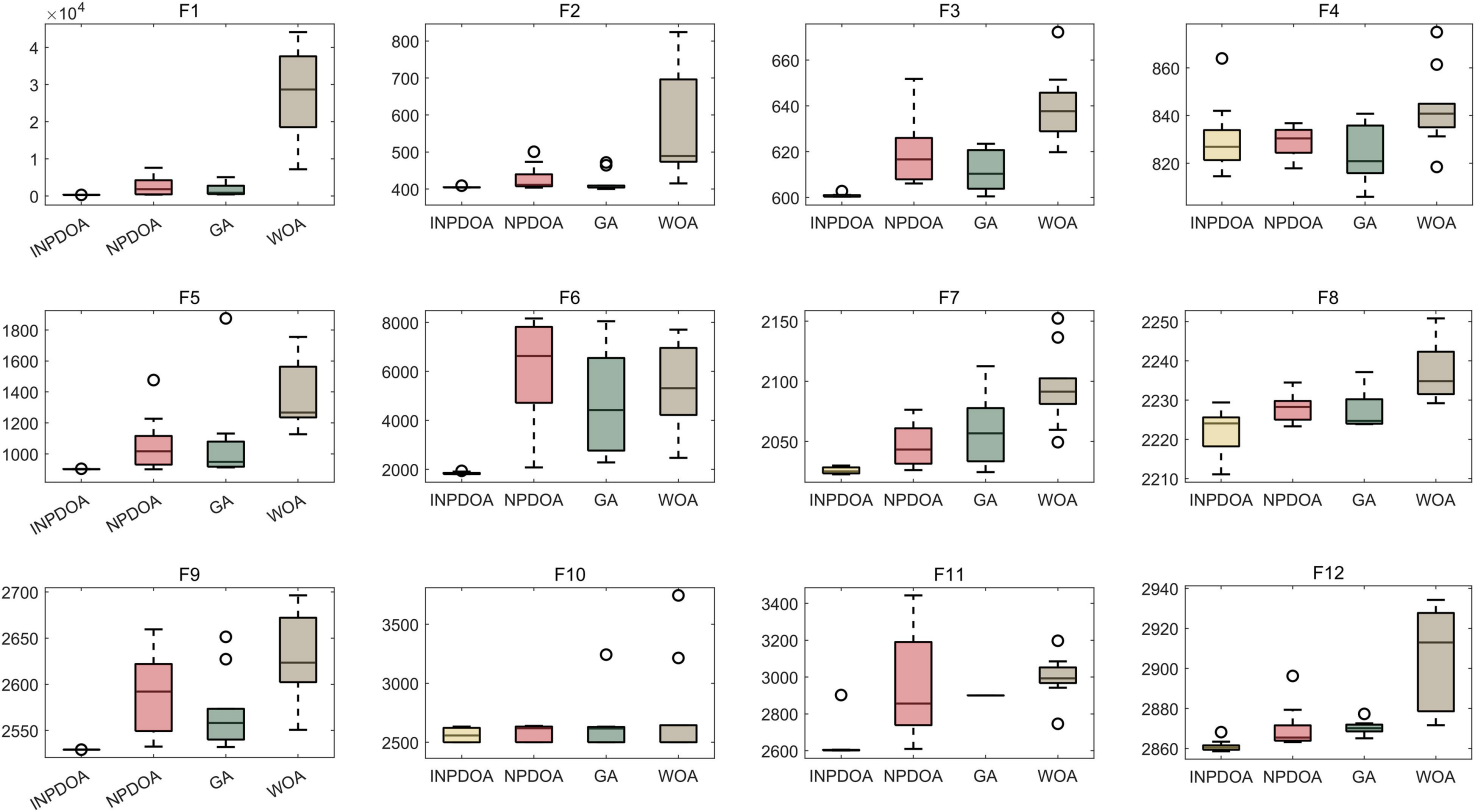
Box plot of algorithm optimization performance comparison.

**Figure 3:**
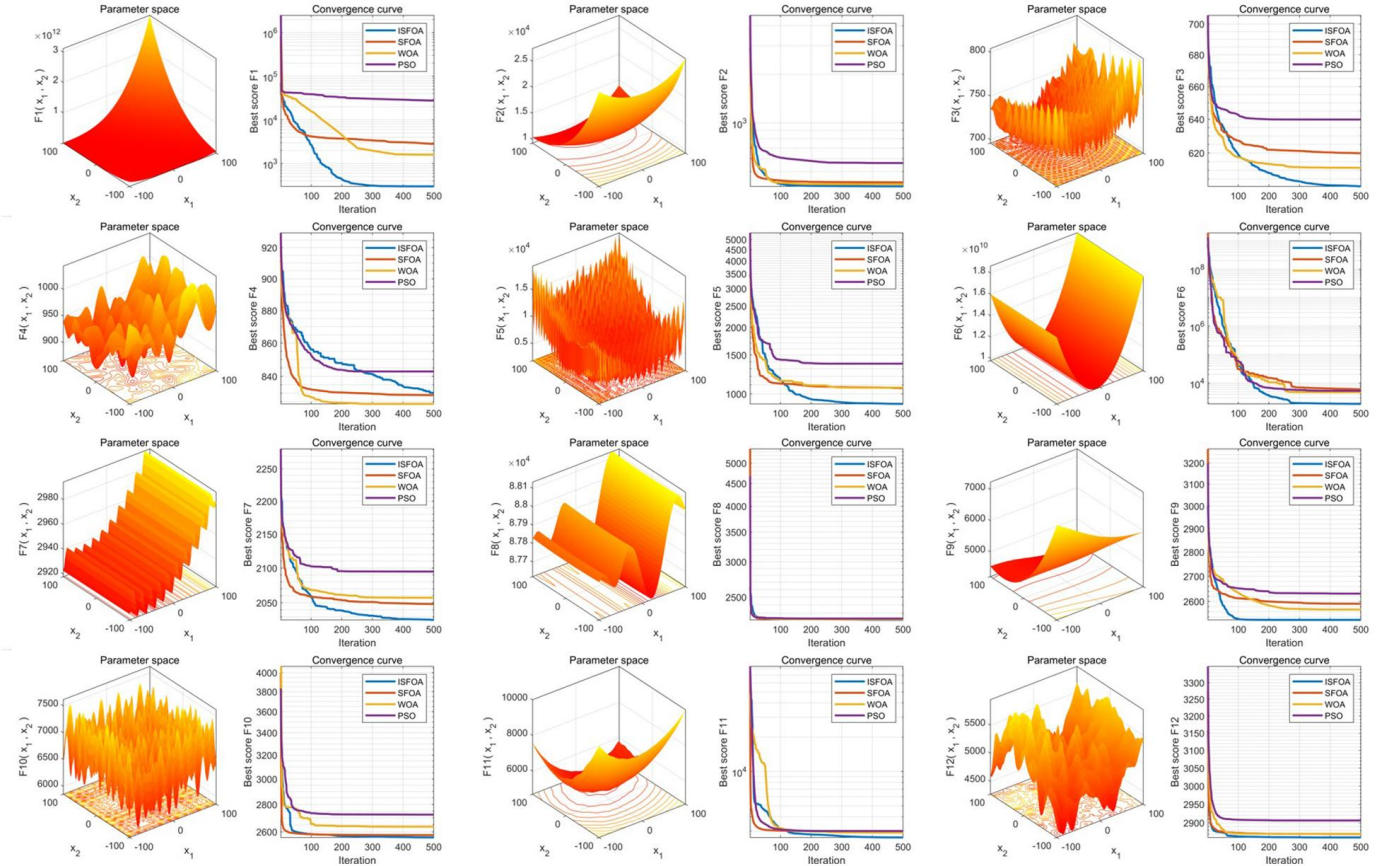
Comparison of convergence performance of the algorithms.

The improved INPDOA demonstrated superior optimization stability and convergence efficiency across 12 CEC2022 benchmark functions (Figures 2, 3). Boxplots of 30 independent runs revealed INPDOA's narrower interquartile ranges (IQRs) and lower outlier frequencies compared to NPDOA, GA, and WOA. Convergence curves highlighted INPDOA's accelerated optimization rates and reduced local optima trapping.

### 3.3 Model training performance

**Figure 4:**
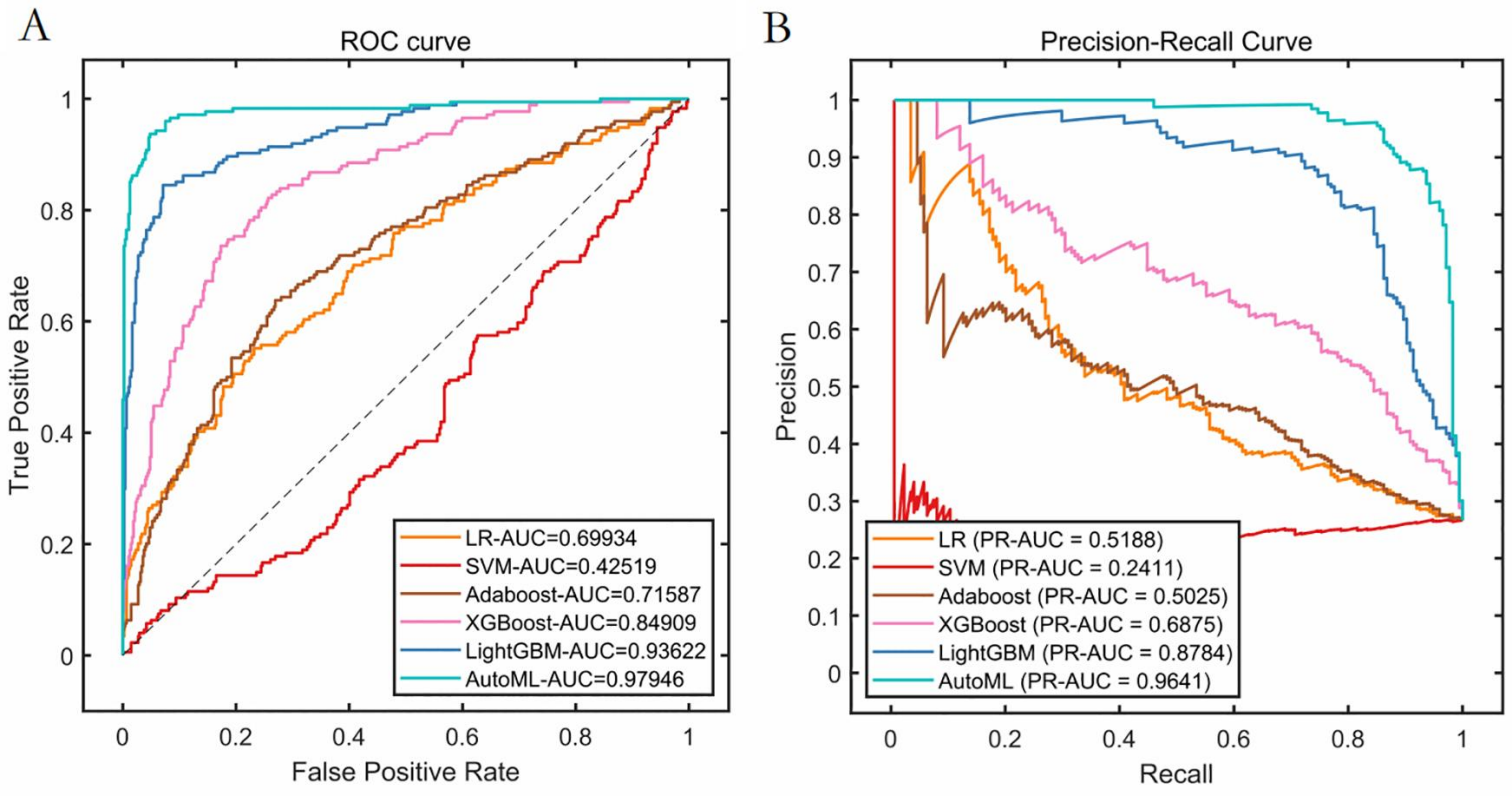
Cross-validation performance performance of the training set. **(A)** ROC curves of the training set. **(B)** PR curves of the training set.

Classification Model (1-month complications): AutoML achieved AUC = 0.9795 and PR-AUC = 0.9641 (training set). Key predictors: Nasal collision within 1 month (SHAP = 0.38), postoperative folliculitis (SHAP = 0.22), animal contact (SHAP = 0.15), spicy food intake (SHAP = 0.13), and smoking (SHAP = 0.12) (Table 2, Figure 4).

**Table 2:** Cross-validation performance of training set (classification model).

Models	PRE	SEN	SPE	ACC	*F*1	ROC-AUC	PR-AUC
LR	0.7037	0.2184	0.9666	0.7672	0.3333	0.6993	0.5188
SVM	—	0.0000	1.0000	0.7335	—	0.4252	0.2411
Adaboost	0.5280	0.3793	0.8768	0.7443	0.4415	0.7158	0.5025
XGBoost	0.8163	0.2299	0.9812	0.7810	0.3587	0.8491	0.6875
LightGBM	0.9787	0.2644	0.9979	0.8025	0.4163	0.9362	0.8784
AutoML	0.9905	0.5977	0.9979	0.8913	0.7455	0.9795	0.9641

The SVM model presented an extreme case and failed to correctly identify any positive samples.

**Figure 5:**
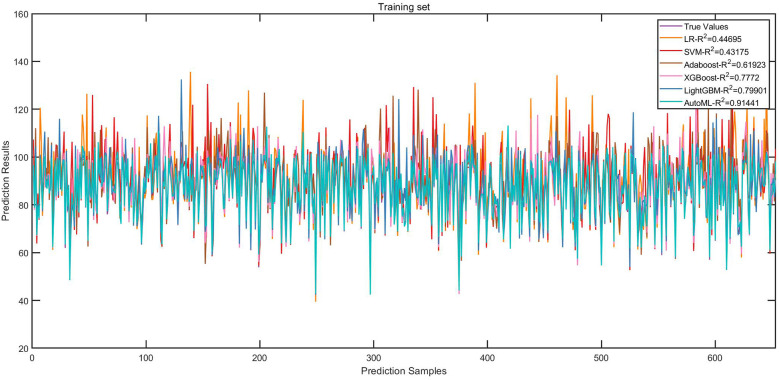
Fitting curves of the training set (regression model).

Regression Model (1-year ROE scores): AutoML yielded *R*^2^ = 0.9144 and MSE = 11.31 (training set). Key predictors: Preoperative ROE score, education level, hospital stay, gender and short-term complications (Table 3, Figure 5).

**Table 3:** Cross-validation performance of training set (regression model).

Models	MSE	RMSE	MAE	*R* ^2^	MAPE
LR	73.1029	8.5500	5.4625	0.4469	6.4950
SVM	75.1116	8.6667	5.3852	0.4318	6.4776
Adaboost	50.3307	7.0944	4.5336	0.6192	5.4428
XGBoost	29.4498	5.4268	3.4190	0.7772	4.0803
LightGBM	26.5672	5.1543	3.2548	0.7990	3.8603
AutoML	11.3138	3.3636	2.1553	0.9144	2.5944

### 3.4 Model validation

**Figure 6:**
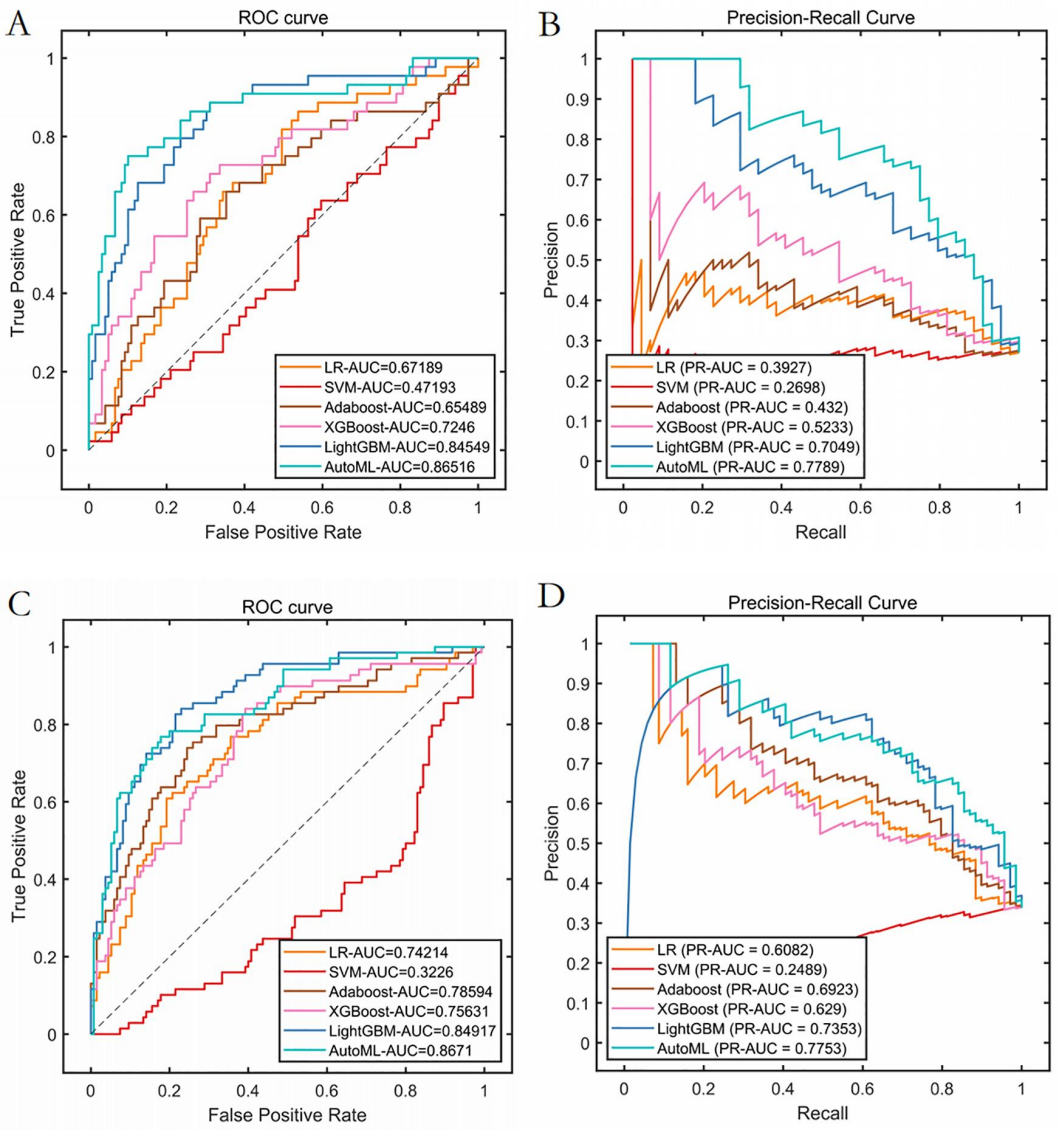
Prediction performance of classification model in test set and validation set. **(A)** ROC curves of the test set. **(B)** PR curves of the test set. **(C)** ROC curves of the validation set. **(D)** PR curves of the validation set.

**Figure 7:**
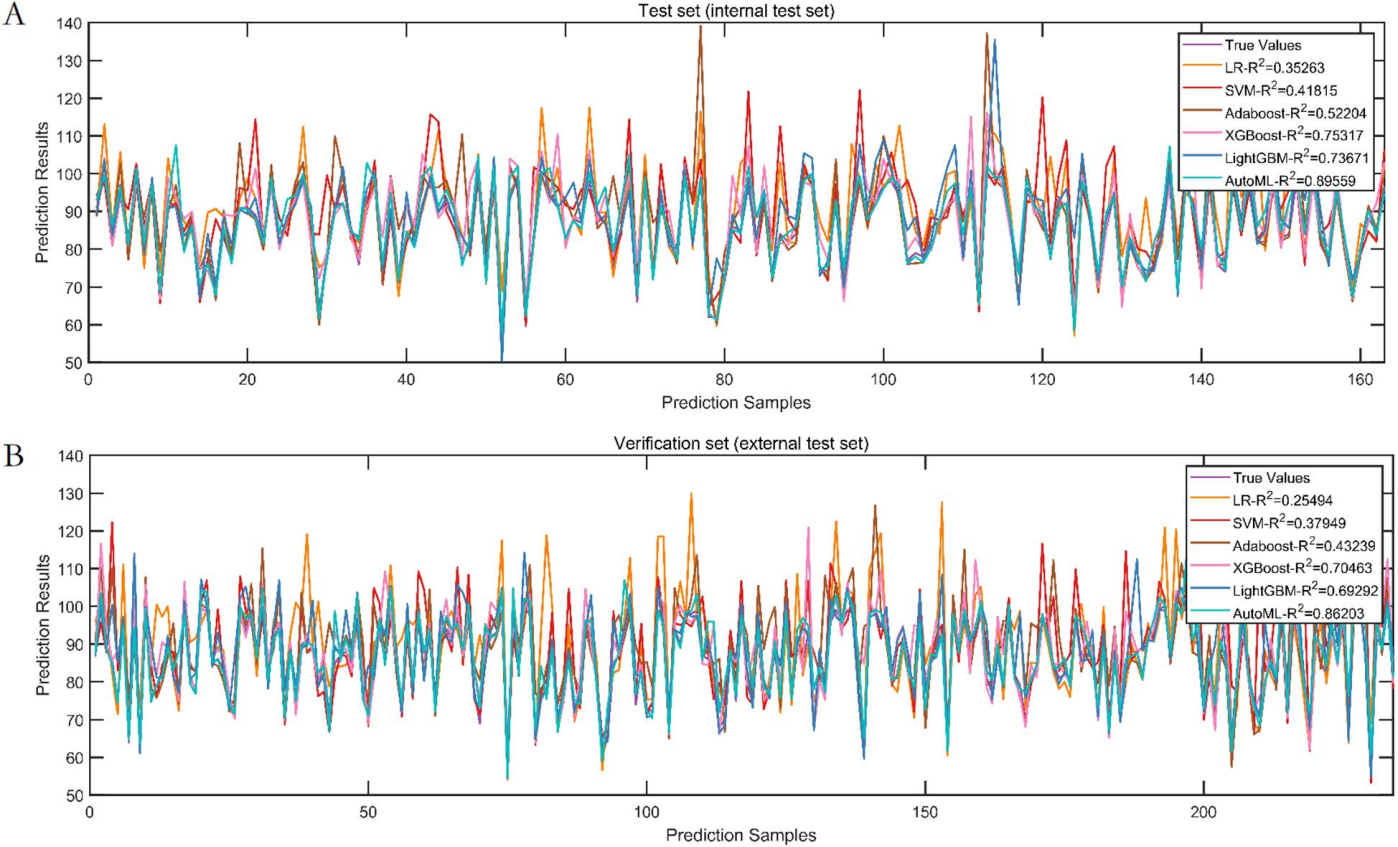
Prediction performance of regression model in test set and validation set. **(A)** Test set. **(B)** Validation set.

Test set: Classification: AUC = 0.8652, PR-AUC = 0.7789, Regression: *R*^2^ = 0.8956, MSE = 12.57; Validation set: Classification: AUC = 0.8671, PR-AUC = 0.7753, Regression: *R*^2^ = 0.8620, MSE = 13.91. Both models significantly outperformed traditional ML approaches (LR, SVM, etc.) in discrimination and calibration (Tables 4, 5, Figures 6, 7).

**Table 4:** Prediction performance of classification model in test set and validation set.

Data set	Models	PRE	SEN	SPE	ACC	*F*1	ROC-AUC	PR-AUC
Test set	LR	0.3000	0.0682	0.9412	0.7055	0.1111	0.6719	0.3927
	SVM	—	0.0000	1.0000	0.7301	—	0.4719	0.2698
	Adaboost	0.5000	0.3409	0.8739	0.7301	0.4054	0.6549	0.4320
	XGBoost	0.6667	0.1818	0.9664	0.7546	0.2857	0.7246	0.5233
	LightGBM	1.0000	0.1364	1.0000	0.7669	0.2400	0.8455	0.7049
	AutoML	0.9333	0.3182	0.9916	0.8098	0.4746	0.8652	0.7789
Validation set	LR	0.7333	0.1594	0.9704	0.6961	0.2619	0.7421	0.6082
	SVM	—	0.0000	1.0000	0.6618	—	0.3226	0.2489
	Adaboost	0.7222	0.3768	0.9259	0.7402	0.4952	0.7859	0.6923
	XGBoost	0.8462	0.1594	0.9852	0.7059	0.2683	0.7563	0.6290
	LightGBM	1.0000	0.0870	1.0000	0.6912	0.1600	0.8492	0.7353
	AutoML	0.9412	0.2319	0.9926	0.7353	0.3721	0.8671	**0** **.** **7753**

The SVM model presented an extreme case and failed to correctly identify any positive samples.

**Table 5:** Prediction performance of regression model in test set and validation set.

Data set	Models	MSE	RMSE	MAE	*R* ^2^	MAPE
Test set	LR	73.4371	8.5695	5.6172	0.3526	6.7638
	SVM	66.0045	8.1243	5.2612	0.4182	6.2461
	Adaboost	54.2200	7.3634	4.2554	0.5220	5.0281
	XGBoost	28.0007	5.2916	3.6727	0.7532	4.3172
	LightGBM	29.8675	5.4651	3.3501	0.7367	3.9943
	AutoML	11.8448	3.4416	2.3054	0.8956	2.7600
Validation set	LR	84.6205	9.1989	6.2201	0.2549	7.3409
	SVM	70.4742	8.3949	5.3124	0.3795	6.3691
	Adaboost	64.4659	8.0291	5.2684	0.4324	6.3697
	XGBoost	33.5472	5.7920	3.7535	0.7046	4.5380
	LightGBM	34.8767	5.9056	3.9302	0.6929	4.6948
	AutoML	15.6697	3.9585	2.5704	0.8620	3.0901

### 3.5 Explainability analysis

**Figure 8:**
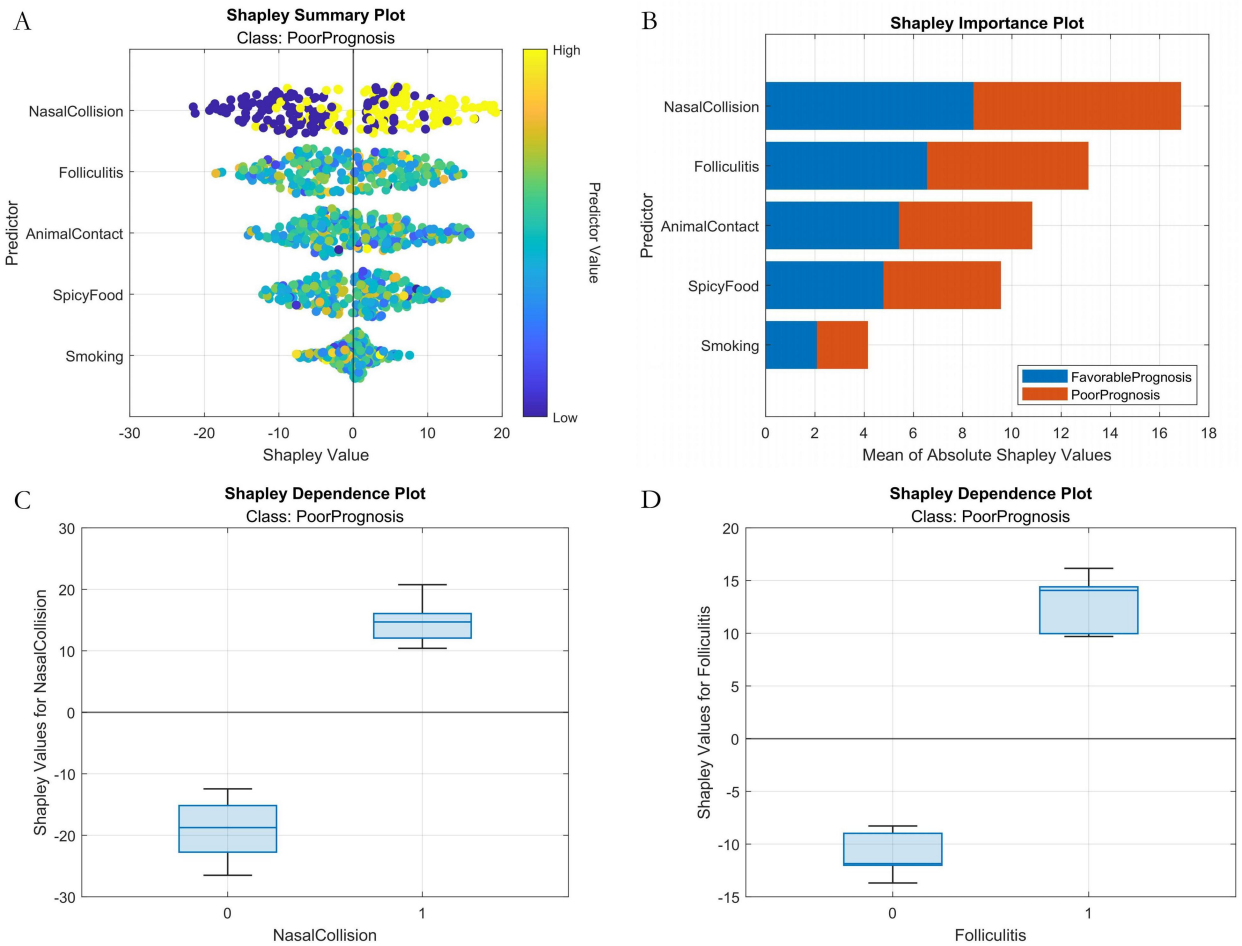
Machine learning interpretability analysis (classification model). **(A)** SHAP summary plot. **(B)** SHAP feature importance bar plot. **(C)** SHAP dependence plot within nasal collision. **(D)** SHAP dependence plot within folliculitis.

**Figure 9:**
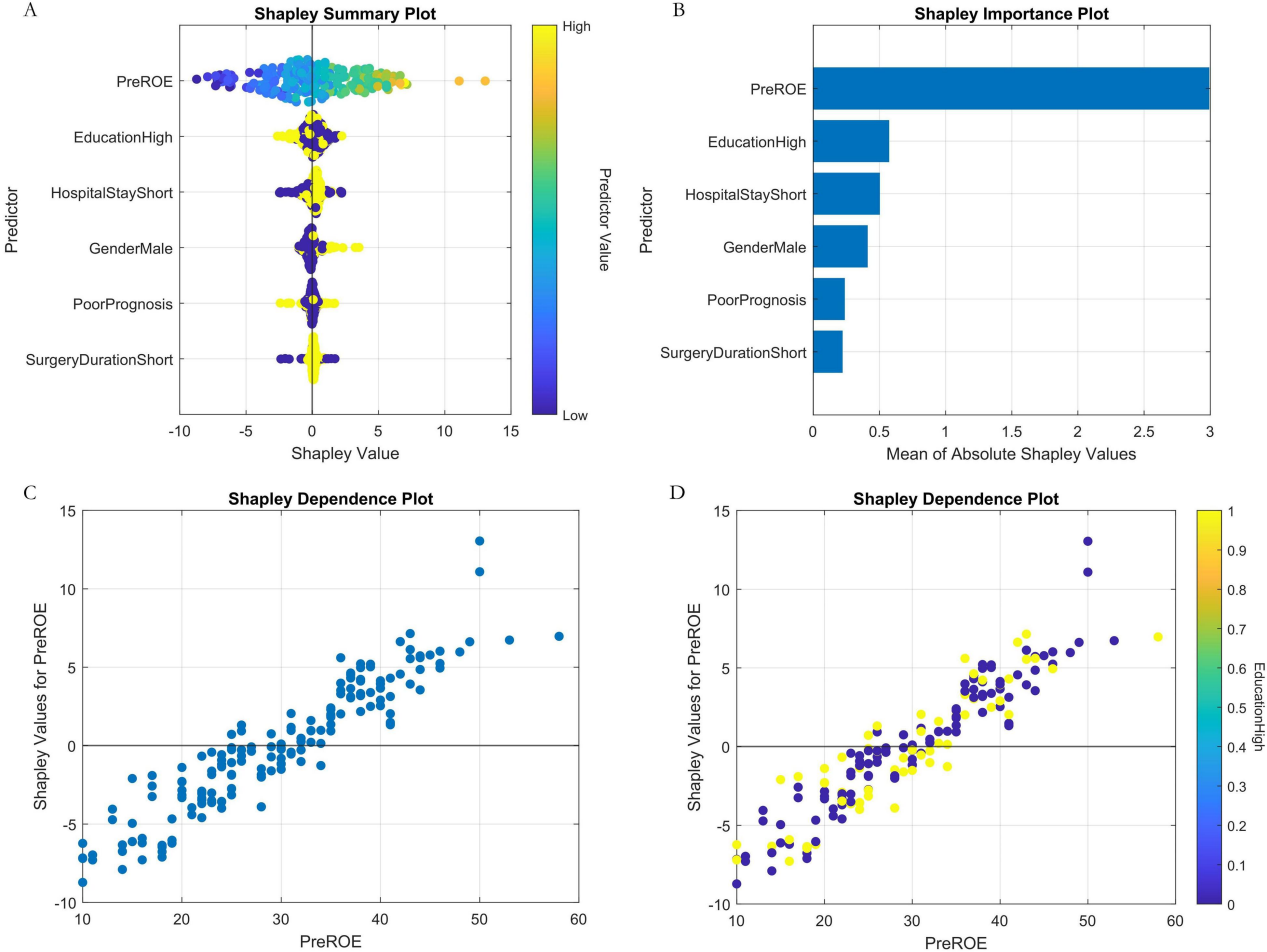
Machine learning interpretability analysis (regression model). **(A)** SHAP summary plot. **(B)** SHAP feature importance bar plot. **(C)** SHAP dependence plot of preoperative ROE score. **(D)** Plot of SHAP interaction dependence between preoperative ROE score and education.

Analysis of the SHAP summary plots (Figures 8, 9) revealed: (1) Classification model: Nasal collision and postoperative nasal folliculitis emerged as core drivers of complication risk (Figures 8A,B). SHAP values exhibited pronounced spikes when these conditions were present (Figures 8C,D), indicating they reached high-risk thresholds. (2) Regression model: Preoperative ROE scores constituted the dominant predictor (Figures 9A,B), demonstrating a critical threshold effect—patients with scores <35 showed reduced long-term prognostic improvement (Figure 9C). Notably, an inverse association existed between ROE scores and education level: the subgroup with low ROE scores exhibited a significantly higher proportion of highly educated patients (>high school education; Figure 9D).

### 3.6 Clinical utility

**Figure 10:**
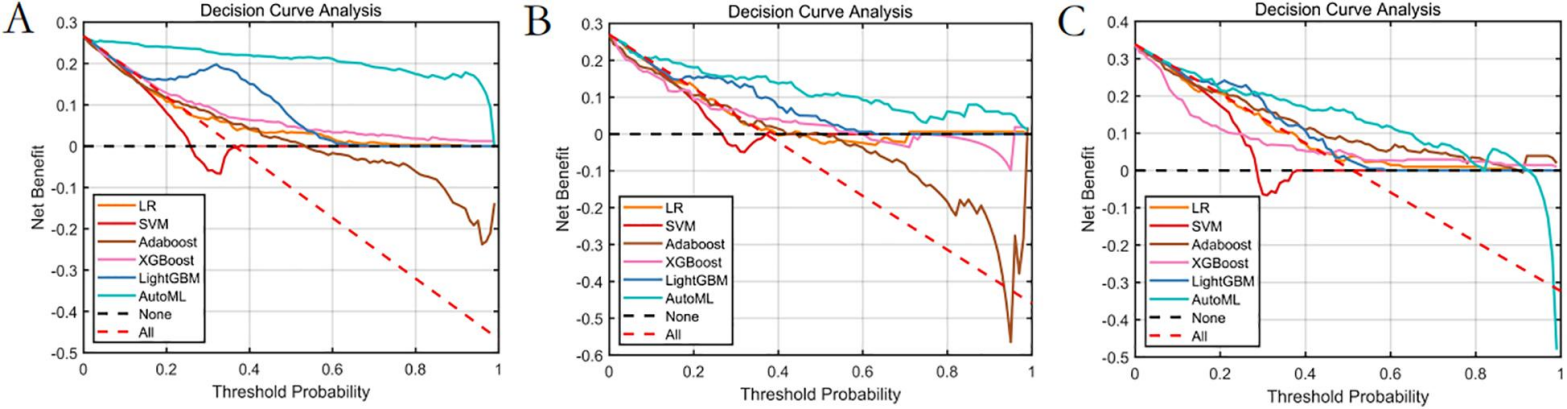
Decision curve analysis of the prediction model. **(A)** Training set. **(B)** Test set. **(C)** Validation set.

**Figure 11:**
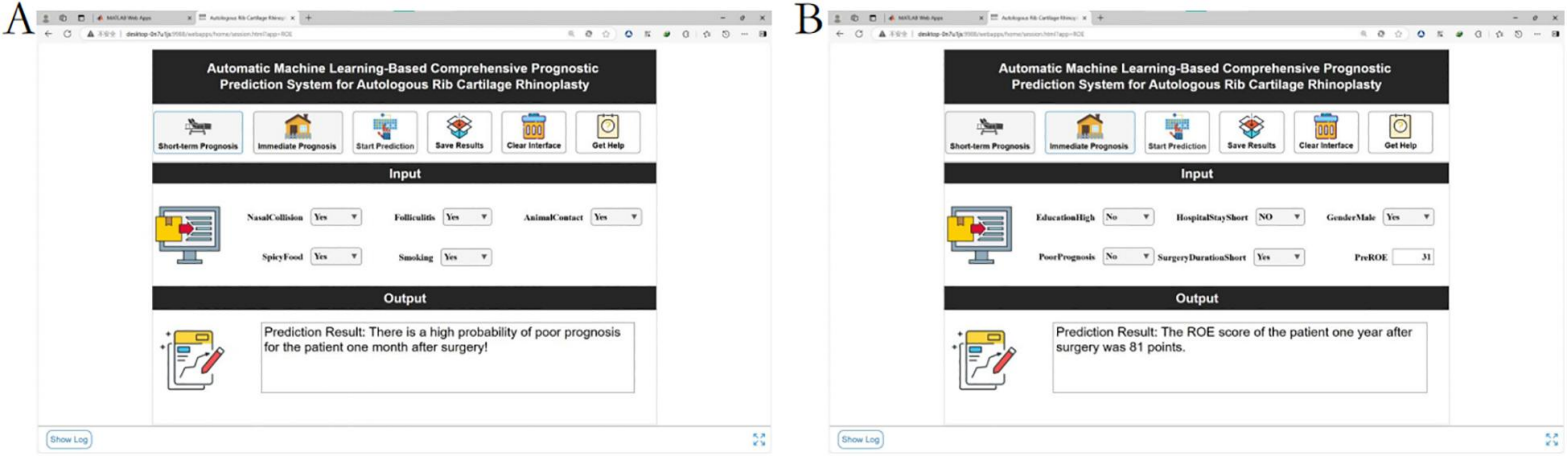
Demonstration of the clinical decision system. **(A)** Short-term outcome prediction: poor prognosis at 1 month after surgery. **(B)** Long-term prognosis prediction: one-year postoperative ROE score.

1. Decision curve analysis (Figure 10): Implementation of the AutoML model yielded a 32% net benefit gain over “treat-all” strategies within a threshold probability range of 10%–50%.
2. Visualization system: A MATLAB-based GUI (Figure 11) achieved <3-second prediction latency with 92% usability satisfaction (surveyed among 15 surgeons). Clinicians input patient parameters via dropdown menus to instantaneously receive risk estimates and evidence-based preventive recommendations.

## 4 Discussion

### 4.1 Core findings and innovative value

Our study established an AutoML-based prognostic prediction system for autologous costal cartilage rhinoplasty (ACCR), with breakthrough innovations in three dimensions: predictive efficacy, clinical application, and methodological advancements. In terms of predictive efficacy, the application of the Improved Neural Population Dynamics Optimization Algorithm (INPDOA) significantly enhanced modeling efficiency. Its global optimization success rate in 12 CEC2022 benchmark tests was markedly higher than genetic algorithm (GA) and whale optimization algorithm (WOA). This breakthrough directly manifested in the leap of model performance: the short-term prognosis classification model achieved an AUC of 0.8671 in the validation set, while the long-term prognosis regression model attained an *R*^2^ value of 0.8620, substantially outperforming the breast reconstruction complication prediction model reported by Naoum et al. (20). This success stems from three technical innovations: (1) Bernoulli mapping initialization strategy enhanced population diversity; (2) Dynamic feature screening mechanism successfully identified high-order interaction effects (e.g., synergistic effects between postoperative nasal collision and smoking) undetected by traditional statistical methods; (3) Explainability design based on SHAP analysis visually presents feature contributions. The clinical value was quantitatively validated through decision curve analysis (DCA), with a net benefit index of 0.32 in the test set, confirming its clinical decision-making improvement significance. As demonstrated in Vickers et al.'s analysis (21), this approach can enhance clinicians' confidence in AI predictions. The developed practical visualization system exhibits intuitive, convenient, and user-friendly advantages.

### 4.2 Paradigm shift compared to traditional prediction systems

Compared to linear prediction models (e.g., CRS-7 scale) used in previous nasal prognosis studies, the performance improvement of our system arises from breakthroughs in three aspects: (1) Data integration: Simultaneous inclusion of biological characteristics (BMI, preoperative ROE score), intraoperative parameters (surgery duration), and postoperative behavioral factors (spicy food intake, animal contact) addresses the limitation of single data sources in prior studies (22). (2) Algorithm architecture: The synergistic application of INPDOA and AutoML improved hyperparameter optimization efficiency. The neural population dynamic update mechanism effectively avoids premature convergence, demonstrating superior global optimization capability compared to particle swarm optimization algorithms reported by Nagra et al. (23). (3) Clinical application: The developed visualization system achieves closed-loop management from prediction to intervention, better aligning with modern medical digital transformation needs than the standalone decision tool designed by Lee et al. (24).

### 4.3 Mechanistic exploration of key risk factors

SHAP analysis revealed that prognostic determinants exhibit significant temporal dynamics and nonlinear associations: (1) Short-term prognosis model: Postoperative nasal collision within 1 month showed the highest SHAP value (0.38, 95% CI: 0.32–0.44), consistent with the biomechanical vulnerability period during early vascularization of autologous cartilage grafts (25). Animal contact history may influence prognosis through two pathways: ① Local immune responses triggered by pet hair promote IL-17 secretion, accelerating graft absorption (26); ② Increased trauma risk elevates microvascular injury probability. Notably, the effect of spicy food intake showed non-monotonic changes: moderate capsaicin consumption (<15 g/day) may counteract inflammatory effects by enhancing blood flow, aligning with Huang et al.'s findings in wound healing (27). (2) Long-term prognosis model: Preoperative ROE score was the top contributing feature, underscoring the central role of psychological expectation management in cosmetic surgery. The negative correlation between male gender and prognosis may relate to skin texture differences—male nasal skin is thinner on average than females (28), increasing graft contour visibility risks. Additionally, reduced satisfaction in patients with surgery durations >8 h may correlate with prolonged cartilage ischemia time and elevated apoptosis rates.

### 4.4 Limitations

Despite significant progress, our study has limitations: ① For AutoML models with over 20 features, the theoretical minimum sample size requirement is 400 cases (20× feature number). Although our sample reached 447 cases, predictive performance might still be affected; ② The validation set only included data from collaborative hospitals in the same region, lacking geographic diversity; ③ Digitized records of intraoperative details (e.g., suturing methods, cartilage cutting angles) risk information loss. These issues represent common challenges in medical AI implementation (29).

### 4.5 Future directions and clinical translation

Building on current results, we will establish a multicenter registry with 5 participating institutions to conduct prospective validation of our AutoML framework. This initiative includes developing HL7 FHIR-compliant interfaces for EHR integration, enabling automatic extraction of predictor variables and embedding of real-time prognostic alerts directly into clinical workflows. Crucially, we are designing patient-facing visualization modules that transform SHAP-derived risk thresholds into interactive decision aids using three key strategies: (1) Risk communication interfaces: Traffic-light visualization of personalized complication probabilities, dynamically updated with mitigation adjustments (e.g., modifying pet contact frequency or smoking cessation targets); (2) Shared decision-making protocols: Co-development of postoperative management plans using AR-powered 3D nasal models superimposed with location-specific risk projections; (3) Threshold-alert system: Automated notifications triggered when EHR-documented variables (e.g., intraoperative duration >8 h) approach high-risk SHAP values. These implementations create a closed-loop framework from prediction to intervention while addressing health literacy disparities through culturally-adapted counseling materials.

## 5 Conclusion

We successfully developed an ACCR prognosis prediction system based on the Improved Neural Population Dynamics Optimization Algorithm (INPDOA) and automated machine learning (AutoML). By integrating multiple features including biological characteristics, predictive models were established. The visualization system supports real-time dynamic predictions, and decision curve analysis (DCA) confirmed increased clinical net benefit. Although sample generalizability requires multicenter validation, this system provides an innovative model for intelligent decision-making in cosmetic surgery, advancing nasal prognosis management into the era of precision medicine.

## Data Availability

The raw data supporting the conclusions of this article will be made available by the authors, without undue reservation.
