# Supplementary material for: Development of a prognostic prediction model and visualization system for autologous costal cartilage rhinoplasty: an automated machine learning approach
Source: Front Surg. 2025 Oct 2;12:1594514. doi: 10.3389/fsurg.2025.1594514 (PMC12528035; doi:10.3389/fsurg.2025.1594514)
Supplement: Supplementary file 1 [file Table1.docx]

**Table S1 Predictor Variables with Clinical Rationale**

| **Category** | **Variable** | **Clinical Rationale** | **Data Collection & Preprocessing** |
| --- | --- | --- | --- |
| **Demographic** | Age | Bone density/skin elasticity decrease with aging; impacts healing time | Continuous values from EMR; converted to integers (years) |
|  |  | Younger patients have higher revision expectations |  |
|  | Sex (Male/Female) | Hormonal differences affect nasal tissue vascular density | Binary coding; validated through ID records |
|  |  | Females show higher complication sensitivity to steroid meds |  |
|  | BMI (kg/m²) | Higher BMI correlates with elevated microvascular complications | Continuous measurement; ±0.1kg precision |
|  |  | Increased adipose tissue in dorsum impacts graft viability |  |
|  | Education Level | Impacts postoperative instruction comprehension | Dichotomized: ≤High school vs >High school |
|  |  | Predicts compliance with nasal splint care (lower=higher displacement risk) |  |
| **Preoperative** | Enlarged Nasal Pores | Sebum hypersecretion increases prosthetic colonization risk | Binary: Y/N by dermatologist evaluation |
|  |  | Predicts early biofilm formation (MSSA/MRSA) |  |
|  | Prior Nasal Surgery | Intranasal scarring reduces tissue perfusion | Verified through surgical logs; binary Y/N |
|  |  | Prior cartilage harvest sites weaken septal support |  |
|  | Preoperative ROE Score | Quantifies baseline functional/aesthetic impairment severity | Continuous (0-100); Rasch-model validated |
|  |  | Lower scores correlate to complex reconstruction needs |  |
| **Surgical** | Hospital Stay (days) | >5-day stays increase multidrug-resistant pathogen exposure | Dichotomized: <5d (early mobilization), ≥5d (prolonged care) |
|  |  | Short stays reduce physiotherapy prep |  |
|  | Surgery Duration (hrs) | >8hr procedures elevate hypothermia/DVT risk | Operating room timestamps; thresholds based on SIRS criteria |
|  |  | Extended exposure promotes airborne contamination |  |
| **Postoperative** | Nasal Trauma (1mo) | Microtrauma disrupts vascular anastomosis sites | Structured questionnaire; binary validation |
|  |  | Primary cause of late hematomas (>7d postop) |  |
|  | Antibiotic Duration | <3d coverage insufficient for porous grafts | Standardized categorization protocol |
|  |  | 3-5d balances SSI prevention vs gut dysbiosis |  |
|  | Folliculitis | Local inflammation initiates graft biofilm cascade | Clinical documentation; photos reviewed |
|  |  | Predictor for early implant explantation |  |
|  | Animal Contact (1mo) | Zoonotic pathogens (Pasteurella spp.) increase necrotizing infection risk | Owner verification; binary reporting |
|  |  | Cat scratches disrupt nasal sutures |  |
|  | Spicy Food (1mo) | Capsaicin triggers histamine release impacting mucosal healing | Diet diary cross-checked with nutritionists |
|  |  | Correlates with epistaxis events |  |
|  | Smoking (1mo) | Nicotine-induced vasospasm reduces flap survival rate (62% vs 94%) | Cotinine urine testing + self-report |
|  |  | Carbon monoxide impairs osteointegration |  |
|  | Alcohol (1mo) | Ethanol metabolites impair fibroblast migration | AUDIT-C scale + pharmacy records |
|  |  | Interaction with perioperative anticoagulants |  |
